# Palliative care needs at a South African district hospital: A descriptive study using supportive and palliative care indicators tool South Africa

**DOI:** 10.4102/jcmsa.v4i1.465

**Published:** 2026-09-30

**Authors:** Louis D.K. Roodt, Cornel F. van Rooyen, Hanneke Brits

**Affiliations:** 1 Department of Family Medicine, School of Clinical Medicine, University of the Free State, Bloemfontein, South Africa; 2 Department of Biostatistics, School of Biomedical Sciences, University of the Free State, Bloemfontein, South Africa

**Keywords:** palliative care needs, end-of-life care, SPICT™-SA, district hospital, primary healthcare, clinical indicators, advanced illness, South Africa

## Abstract

**Background:**

Patients with advanced life-limiting diseases (ALLDs) are often admitted to district hospitals for acute curative care, resulting in delayed or omitted palliative care. Early identification by clinicians ensured better care, efficient resource use and prevented avoidable hospitalisations. This study aimed to determine the prevalence and profile of admitted adult patients with palliative care needs at National District Hospital (NDH).

**Methods:**

This was a retrospective descriptive study. Investigators identified palliative care needs based on the Supportive and Palliative Care Indicators Tool South Africa (Supportive and Palliative Care Indicators Tool [SPICT]™-SA) criteria.

**Results:**

Out of 297 patient records reviewed, 127 (42.8%) met the criteria for palliative care based on SPICT™-SA. The most common qualifying disease-specific diagnoses included infectious diseases (21.8%), neurological conditions (15.6%), dementia or frailty (13.6%), and kidney disease (15.0%), while cancer (including haematological malignancies) accounted for 10.9%. The general indicators qualifying for palliative care included frequent unplanned visits (39.3%), poor functional status (26.2%) and dependence on others for care (24.3%).

**Conclusion:**

The findings pointed to a gap in recognising palliative care needs among hospitalised patients, especially those with non-malignant conditions. Introducing routine screening using the SPICT™-SA tool at the district level supported earlier identification and enabled planning of care. This led to improved patient outcomes and effective use of health system resources.

**Contribution:**

The study demonstrated how routine screening with the SPICT™-SA tool during admission could help identify palliative care needs in a district hospital.

## Introduction

Palliative care is an important aspect of comprehensive healthcare, providing a multidisciplinary approach to enhance the quality of life for patients and their families facing life-limiting diseases.^1^ Resolution 67.19 of the World Health Assembly emphasises that palliative care is an ethical obligation of health systems. It emphasises the responsibility of healthcare providers to alleviate pain and suffering.^2^ Although healthcare systems increasingly recognise palliative care as a fundamental component of comprehensive care, many resource-limited settings continue to under-prioritise it.^3,4^ In South Africa, only 18% of adults and 5% of children who require palliative care receive this indispensable service.^5^ The National Policy Framework and Strategy on Palliative Care (NPFSPC) estimated that nearly half of all deaths in 2014 required some form of palliative care; yet, accessibility remains a challenge because of under-resourced systems and high disease burdens.^6^

One of the greatest obstacles in care delivery is the persistent failure to recognise palliative care needs among patients with non-cancer conditions.^7^ While clinicians have traditionally linked palliative care with oncology, growing evidence highlights that patients with cardiovascular diseases, chronic respiratory diseases, diabetes and infectious diseases such as Human Immunodeficiency Virus (HIV) and tuberculosis (TB) have equally profound needs.^7^ Yet, the national guidelines and hospital-based palliative care services tend to prioritise cancer-related needs, leading to missed opportunities for timely intervention in those with organ failure and infectious diseases.^7^ Furthermore, there is currently limited research on the prevalence and profile of patients requiring palliative care at the district hospital level in South Africa, as most data are focused on secondary or tertiary settings. This study attempted to address this knowledge gap by exploring these specific needs within a district hospital environment where clinical leadership and standardised training are often lacking.

A significant challenge in palliative care delivery is the timely identification of patients who stand to benefit from palliative interventions.^6^ This study is underpinned by the premise that objective clinical criteria are preferred over subjective judgement when determining care needs, as objective criteria minimise bias and improve diagnostic accuracy.^3^ The use of standardised screening tools, such as the Supportive and Palliative Care Indicators Tool (SPICT™), which is an internationally validated screening tool, at a district level hospital, could enhance the early recognition and management of patients with unmet palliative care needs.^8,9,10^ While SPICT™ acknowledges the uncertainty of prognosis, its primary objective is to ensure that patients with advanced life-limiting diseases (ALLDs) receive timely palliative care interventions, reducing unnecessary delays in care planning. However, we should recognise that screening tools developed in high-income countries may not fully capture the healthcare realities of South Africa. Therefore, this study utilised a locally adapted SPICT™-SA, to better reflect the national disease burden.^11^ South Africa faces a unique prevalence of infectious diseases such as HIV and TB, as well as trauma-related conditions, factors that are frequently underrepresented in internationally developed palliative care tools. To address these contextual differences, SPICT™-SA was created through a Delphi study to ensure both clinical and cultural relevance within the South African healthcare setting.^11^

The aim of this study was to determine the prevalence and profile of adult patients admitted to National District Hospital (NDH) with palliative care needs. To achieve this, the study identified three primary objectives: (1) identifying patients admitted to the adult wards at NDH with palliative care needs; (2) calculating the actual prevalence of patients meeting these criteria; and (3) describing their clinical and sociodemographic profiles to better understand the population requiring these essential services.

## Research methods and design

### Study design

This project utilised a retrospective descriptive study design to evaluate the prevalence and clinical profile of patients within the adult wards.

### Setting

National District Hospital is a public district hospital located in Bloemfontein, within the Mangaung Metropolitan Municipality of the Free State province. It functions as both an academic training facility and a level-one hospital. Operating within a larger referral network, NDH works in collaboration with Pelonomi Tertiary Hospital and Universitas Academic Hospital, facilitating both the referral of critically ill patients and the management of down-referrals. With 197 inpatient beds, the hospital serves a catchment area of approximately 598 000 residents and acts as the referral centre for two community health centres and 15 primary healthcare clinics.^12^

The facility provides a broad range of services, including outpatient, emergency, inpatient (medical, surgical, paediatric, and maternity), and theatre services. While these encompass diagnostic, curative, and supportive care, the hospital faces a significant clinical burden. In the adult medical wards alone, NDH averages 310 admissions and 60 deaths per month.^13^ Between April 2022 and March 2023, the hospital recorded 3921 admissions and 852 deaths, with the majority (620) occurring in the wards and 232 in the emergency department.^14^ The leading causes of mortality during this period included pneumonia, TB, renal failure, cardiovascular accidents, and HIV.^13^ Crucially, despite this high mortality rate, NDH still lacks a structured palliative care protocol to guide clinicians in identifying and managing patients with palliative needs.

### Study population and sampling strategy

The study included all adult patients aged 18 years and older who were admitted to the medical wards at NDH. With approximately 4000 annual admissions, a systematic sampling approach was utilised by including all patients admitted during three distinct periods in 2023: January, April, and July. These specific months were selected to ensure a representative sample and mitigate potential seasonal bias in disease presentation. It was anticipated that this timeframe would yield between 800 and 1000 patient files. To ensure a comprehensive overview of the hospital’s clinical load, the investigators included patients from all four adult medical wards.

### Data collection and measurement

The investigators utilised the SPICT™-SA to screen all eligible patient records. The SPICT™-SA is a standardised clinical tool designed to help practitioners identify people living with one or more ALLDs. It consists of two main sections: (1) general indicators of deteriorating health (such as unplanned hospitalisations or declining performance status); and (2) disease-specific indicators (including respiratory, kidney, or neurological diseases, and specifically HIV and TB for the South African context).^11^

For this study, a patient was identified as having palliative care needs if they met at least two of the general indicators alongside one or more disease-specific indicators. To ensure data integrity, all information from the physical hospital files was transferred by the researcher into a secure, password-protected Redcap™ database hosted by the University of the Free State (UFS). This process involved capturing sociodemographic data, admission details, and the specific SPICT™-SA clinical indicators present at the time of the hospital encounter.

### Pilot study

The investigators conducted a pilot study in January 2024 to test the data extraction process and the applicability of the SPICT™-SA tool. During this phase, it was discovered that the files from January 2023 were inaccessible because of a system-wide change in filing and numbering protocols at the facility. Consequently, the investigators refined the sampling strategy to focus exclusively on the months of April and July 2023. This adjustment ensured that the data utilised for the final analysis was complete and consistent across the study period.

### Data analysis

Data were captured on the *Protection of Personal Information Act* (POPIA)-compliant REDCap platform hosted on the UFS’s secure server. The Department of Biostatistics performed statistical analysis using the Statistical Analysis Software (SAS version 9.4) (Institute Inc., Cary, North Carolina, United States). Descriptive statistics, including frequencies and percentages for categorical variables and means and medians for numerical data, were utilised to summarise the sociodemographic and clinical profiles of the patients. The prevalence of palliative care needs was calculated as a percentage of the total sample retrieved.

Because this was a retrospective record review, a waiver of individual informed consent was requested and formally granted by the Health Sciences Research Ethics Committee (HSREC). To ensure patient confidentiality and data protection, all extracted information was anonymised by the primary investigator at the point of collection. No identifiable patient details were recorded in the study database. Data were stored securely on the password-protected Redcap™ platform, accessible only to the research team and the biostatistician. The original, de-identified dataset will be securely stored for a minimum of 5 years from the publication date. Following this period, the authors will responsibly dispose of the project’s data.

### Ethical considerations

The study was conducted in accordance with the Declaration of Helsinki. Full ethical approval was obtained from the Health Science Research Ethics Committee of the University of the Free State on 09 November 2023 (Approval no: UFS-HSD2023/0943/3001). Institutional permission was also granted by the head of the Free State Department of Health.

## Results

The investigator identified a total of 1036 patient files from the ward registers for the study period. As observed in the pilot study, 362 files from January were excluded due to changes in the filing system that rendered them irretrievable. Of the remaining 674 files from April and July, 300 (44.5%) were successfully retrieved, although three were found to be empty and excluded from the analysis. Only 127 files met the SPICT^TM^-SA criteria of the study (See Figure 1 for the breakdown).

**FIGURE 1:**
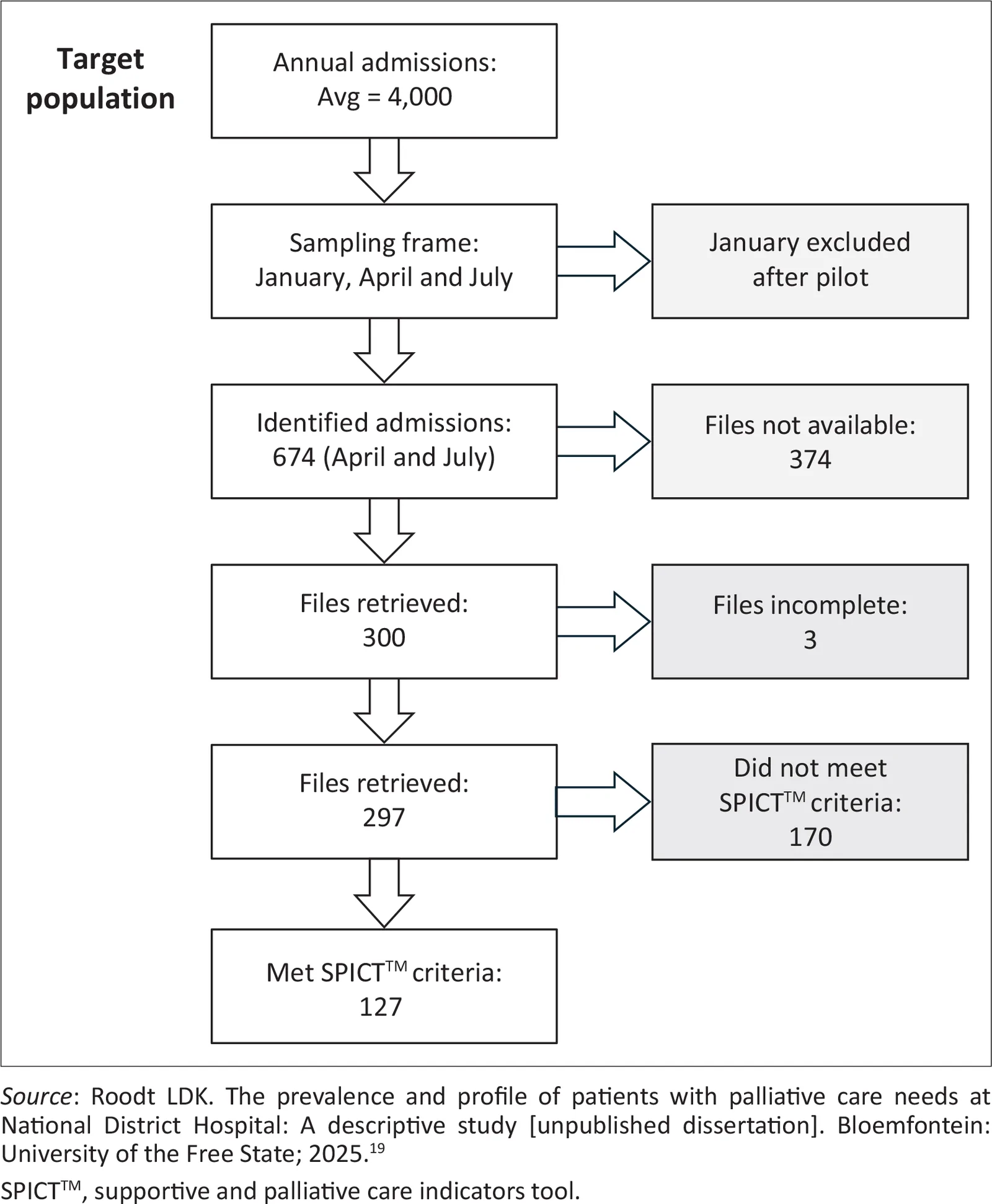
Flowchart of sample selection.

The remaining results apply to patients with palliative care needs identified by the SPICT™-SA tool.

### The prevalence of palliative care needs at National District Hospital

Of the adult patients admitted during the study period, 42.8% met the SPICT™-SA criteria for palliative care needs. The distribution of SPICT™-SA disease categories showed that infectious diseases (21.8%) were the most common condition qualifying for palliative care, followed closely by neurological diseases (15.6%) and kidney disease (15%), while cancer (including haematological malignancies) accounted for 10.9%. Non-malignant haematological diseases accounted for 0.7%. The complete distribution of clinical categories is detailed in Figure 2.

**FIGURE 2:**
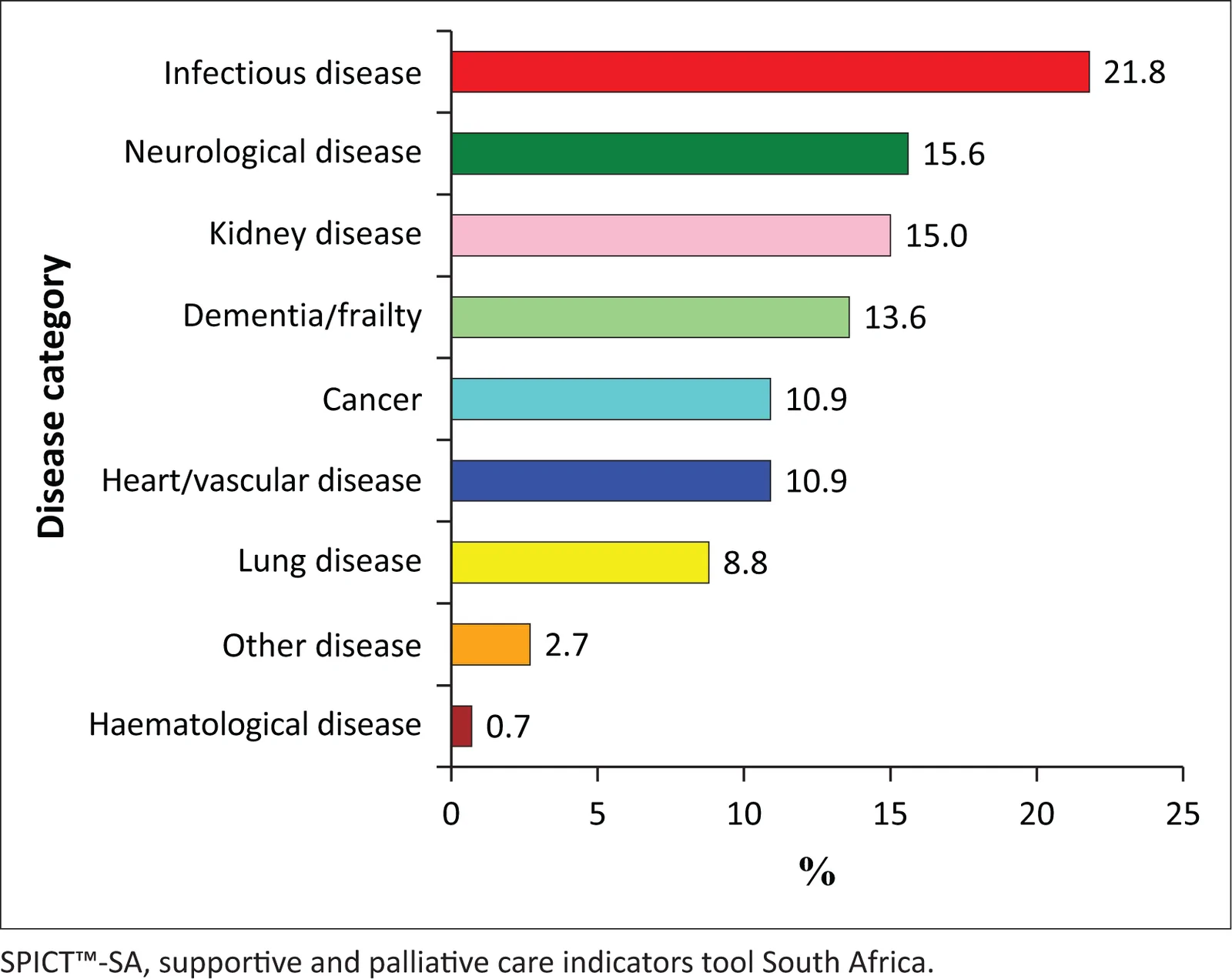
Percentage of patients according to SPICT™-SA disease categories.

Analysis of the SPICT™-SA general indicators revealed that frequent, unplanned healthcare visits (39.3%) were the most common sign of health deterioration, followed by poor performance status and functional dependence. Notably, no subjective requests for palliative care from patients or families were recorded. Detailed frequencies for all general indicators are provided in Figure 3.

**FIGURE 3:**
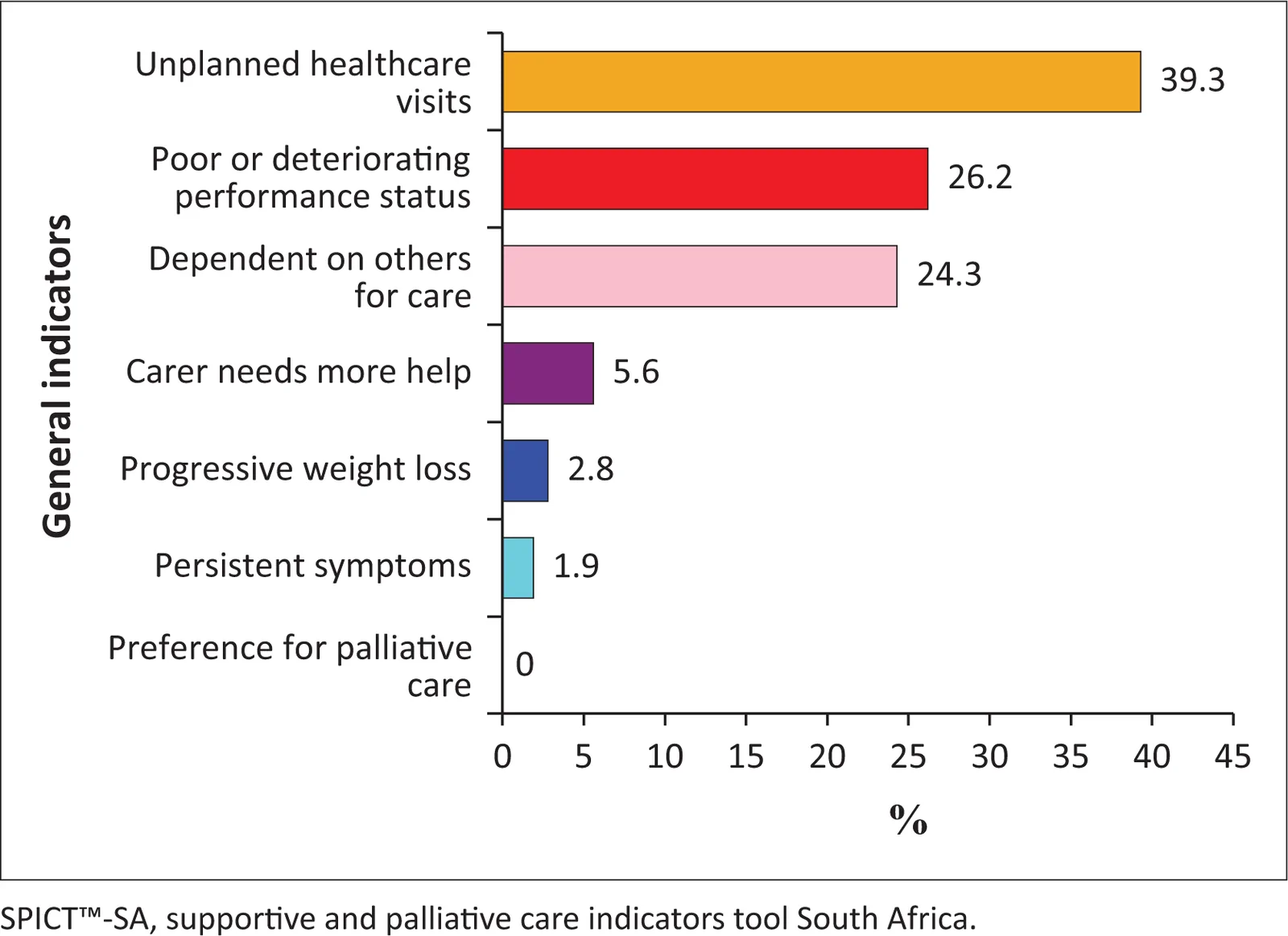
Percentage of patients with SPICT™-SA general indicators.

### Demographic and clinical characteristics

The study population (*n* = 127) showed a nearly equal gender distribution, with a slight male predominance (51.9%). While the sample spanned various age groups, nearly half were older than 60 years (45.6%), representing the largest single demographic requiring palliative intervention. Geographically, most (81.1%) were residents from the Mangaung district (Table 1).

**TABLE 1:** Sociodemographic and clinical characteristics of the study population (*N* = 127).

Characteristic	*n*	%
**Sex**		
Female	61	48.0
Male	66	51.9
**Age (years)**		
18–27	4	3.1
28–38	16	12.6
39–49	26	20.4
50–60	23	18.1
> 60	58	45.6
**Triage code**		
Green	0	0.0
Yellow	23	18.1
Orange	52	40.9
Red	9	7.1
Unknown	43	33.8
**District**		
Mangaung	103	81.1
Other	24	18.9
**Referral**		
Family	38	29.9
Health institutions	35	27.6
Unknown	29	22.8
Self	22	17.3
Spouse or partner	5	3.9
Friend	3	2.4
**Hospital stay (days)**		
0–2	4	3.3
2–5	30	24.2
> 5	92	72.5
**Outcome**		
Discharged	88	69.5
Referred	20	16.1
Deceased (10 male and 8 female)	18	14.4

Most patients were triaged as orange (40.9%), while the triage code was unknown for 33.8%. Referral patterns were diverse, but most patients were brought in by family members (29.9%). The length of hospital stay was more than 5 days in 72.5% of cases, 2–5 days in 24.2% and 0–2 days in 3.3%. While the majority (69.5%) recovered sufficiently to be discharged home, the in-hospital mortality rate was 14.4% (*n* = 18). The leading causes of death were pneumonia, TB, renal failure, congestive cardiac failure and meningitis.

## Discussion

This study reveals valuable insights into the prevalence and profile of adult inpatients with palliative care needs at NDH. The results suggest that nearly half of adult admissions met the SPICT™-SA criteria. However, this is limited by the low retrieval rate of patient files, which is further discussed in the section on limitations.

The study found that 42.8% of adult inpatients met SPICT™-SA criteria for palliative care needs. This is substantially higher than previous local studies, such as the 2014 Cape Town study, which reported a 16.6% prevalence using less inclusive criteria.^15^ However, the high prevalence must be interpreted with caution. Retrospective data extraction relies on the quality of original clinical notes. Parameters such as poor functional status or dependence on others were inferred from nursing assessments and physiotherapy notes, which may lead to subjective overestimation or underestimation because of missing data.

National District Hospital functions as both a primary and referral hospital. It receives 27.6% as down-referrals from tertiary centres, contributing to the higher prevalence of palliative care needs. These patients often present with advanced disease, increasing the likelihood of needing palliative care. The lack of clear national palliative care guidelines for infectious diseases, such as HIV and TB, may also complicate timely identification and contribute to under-recognition at earlier stages. The inclusive screening strategy may have also contributed to a higher rate, potentially including patients without immediate palliative care needs.

The SPICT™-SA tool effectively identified a range of disease-specific indicators: infectious diseases were the most common (21.8%), aligning with South Africa’s high burden of HIV and TB. The high burden of infectious diseases contrasts with findings from Van Niekerk and Raubenheimer, where only 9.6% of palliative care-eligible patients had HIV and TB, and 50.8% had cancer.^15^

This study identified cancer (including haematological malignancies) in only 10.9% of cases, reinforcing the growing recognition that palliative care extends beyond the realm of oncology. Neurological diseases (15.6%) and dementia and frailty (13.6%) were also prominent, supporting the need for broader multidisciplinary services that include neurodegenerative and age-related conditions. Of note, the researcher identified no patients with trauma-related palliative care needs, likely reflecting NDH’s referral protocols, as they usually refer trauma cases to Pelonomi trauma unit. The low proportion of non-malignant haematological conditions (0.7%) and cancers (10.9%) may reflect effective existing palliative care plans for these subgroups, resulting in fewer acute hospital presentations, rather than merely under-detection.

The General SPICT™-SA indicators revealed high levels of health system utilisation and functional decline. Of all visits, 39.3% were unplanned, and this was the most met criterion. This highlights the fact that opportunities are often missed for outpatient symptom management or advanced care planning. Progressive functional decline and high caregiver burden were evident by deteriorating performance status (26.2%) and dependence on others for daily care (24.3%). However, documentation of caregiver burden (5.6%) was limited, suggesting possible under-recognition of family strain. The clinical deterioration markers, such as weight loss (2.8%) and persistent symptoms despite treatment (1.9%), were under-recorded; this reflects potential gaps in routine symptom assessment. Notably, no patients explicitly requested palliative care, suggesting low awareness or acceptance of palliative care as a treatment pathway.

Patient profile data further contextualises these findings. The most common referral source was family-driven (29.9%), indicating that patients’ families continue to seek curative services. This finding aligns with existing literature, which indicates that insufficient community-based palliative care services often lead patients and their families to seek hospital-based care, thereby increasing the burden on emergency departments and resulting in delayed palliative care interventions.^16^

Health-institution referrals (27.6%) were predominantly for continued curative care rather than palliative needs, underscoring a systemic gap in recognising palliative care-eligible patients during routine care transitions. Self-referral (17.3%) and undocumented referral sources (22.8%) further indicate the absence of structured palliative care referral pathways.

Triage data revealed a discrepancy between emergency care classification systems and the realities of palliative care. Clinicians triaged most patients as urgent (40.9%) or moderate (18.1%) and none as non-urgent. A total of 28.4% of files lacked documented triage codes, which may reflect documentation issues and a misalignment between the Triage Early Warning Score (TEWS) and the nature of palliative diseases. Triage Early Warning Score, which was designed for acute deterioration, may fail to identify patients with chronic progressive decline. This misclassification may contribute to overtreatment, unnecessary admissions and inappropriate allocation of emergency resources, indicating the need for more suitable triage tools for palliative care populations.

The demographic profile of patients with palliative care needs offers additional insights. The median age was 58 years, which closely aligned with the mean age of 56 years reported by Van Niekerk and Raubenheimer.^15^ This is below South Africa’s national life expectancies of 65.5 years for men and 71.0 for women.^17^ The gender distribution was nearly equal (52.0% male vs. 48.0% female), although this differs from the general hospital admission patterns at NDH, where female patients represent 59% of all admissions at NDH.^13^

This gender balance underscores the importance of providing equitable and gender-sensitive palliative care services. Most patients (81.1%) were from the Mangaung district, confirming NDH’s role as both a local and regional referral centre for palliative care.

Patient outcomes reveal further concerns. Most patients (69.5%) were discharged, indicating that despite meeting palliative care criteria, they may not have received structured palliative care support. Discharge without coordinated care planning leads to unmet symptom needs, carer strain and readmissions. Clinicians referred 16.1% for further care, solely for ongoing curative care. The inward mortality rate (14.4%), with common causes including pneumonia, TB, renal failure, and cardiac disease, reflects late presentation and missed opportunities for early intervention. During the study period in April and July 2023, NDH’s adult inpatient mortality rate was 14.4%, closely aligning with the hospital’s mortality rate of 15.8% reported between April 2022 and March 2023.^13^ This is consistent with the findings from a Western Cape study, which estimated that 14.0% of admitted patients were expected to die within 1 year, highlighting the ongoing need for structured palliative care services.^18^

Finally, admission duration reflects the resource-intensive nature of care. Over 70% of patients stayed longer than 5 days, often receiving investigations and treatments of questionable benefit given their disease trajectories. Prolonged admissions suggest a continued curative focus and highlight the need to align hospital care with patient-centred palliative goals. Improved documentation, routine screening, and referral to community-based palliative care services could reduce unnecessary hospital stays while improving quality of life.

### Strengths and limitations

#### Strengths

This study is the first to assess palliative care needs at a district hospital level in South Africa using the SPICT™-SA tool. It addresses a critical gap in local research by identifying palliative care needs beyond oncology, particularly among patients with infectious diseases, neurological conditions and chronic organ failure. The study offers a starting point for integrating palliative care into routine workflows within district hospitals.

Although researchers derived the SPICT™-SA tool from a widely accepted international framework, it has not yet been formally validated for the South African context. Nevertheless, its structured format proved feasible and easy to implement in this setting, supporting the identification of patients who may benefit from palliative care despite limited specialist resources. The findings suggest that with appropriate adaptation, SPICT™-SA may be a useful clinical tool in similar environments.

The district hospital context enhances the study’s relevance for resource-limited public healthcare facilities, where palliative care services are often underdeveloped. The research provides insight into the types of life-limiting illnesses presenting at this level of care. It highlights the operational gaps in referral patterns, triage and symptom documentation.

#### Limitations

The author considered several limitations, including a low file retrieval rate of 44.0%, which was primarily because of the inefficiency of manual record-keeping systems. Misfiled, missing or lost patient files limited the study’s data quality and posed broader clinical risks, including disrupted continuity of care, delayed referrals and compromised clinical decision-making.

In addition, the inability to access all eligible files may have introduced selection bias, especially if missing records belonged to patients with more complex or severe illness trajectories. Conversely, the prevalence of palliative care needs could have been overestimated if the missing records were predominantly from patients with acute, curable medical conditions.

Conducting the study in a single facility limits the ability to generalise the findings to other districts or tertiary hospitals with different patient populations or healthcare systems. Differences in infrastructure, referral networks and disease burdens may impact the prevalence and nature of palliative care needs in other settings.

### Implications and recommendations

The findings support the inclusion of SPICT™-SA screening as part of routine admission processes. Doing so could help clinicians identify patients who are unlikely to benefit from curative treatment alone and may require a palliative approach instead.

The high rate of family-driven referrals highlights a reactive approach to palliative care rather than structured clinician-led identification. Standardised referral protocols should be developed and embedded into routine ward-based clinical workflows to address this.

Improved symptom documentation and clinical assessment are needed. Key indicators such as weight loss and persistent symptoms were frequently under-recorded, suggesting that healthcare providers would benefit from focused training in recognising and documenting palliative care needs.

The current triage system, designed primarily for acute emergencies, does not reliably identify patients with progressive functional decline. The hospital should consider adapting the triage tools to reflect the nature of palliative disease and guide appropriate referrals.

## Conclusion

This study demonstrated that a significant proportion of adult inpatients at NDH have unmet palliative care needs by using the SPICT™-SA tool. The SPICT™-SA tool successfully identified patients with potential palliative care needs, achieving the study’s objective of screening admitted patients for advanced illness. The prevalence demonstrates that many hospitalised adults may benefit from timely palliative care interventions. Furthermore, the study described the profile of this population, highlighting that most patients were middle-aged adults, presented with non-cancer conditions such as infectious and neurological diseases, and experienced poor functional status and prolonged hospitalisation. These findings suggest that structured palliative care is underutilised and often never introduced during hospital admissions. Supportive and Palliative Care Indicators Tool South Africa offers a practical solution to support earlier identification, guide appropriate referral and inform discharge planning.
